# LncRNA TP53TG1 Promotes the Growth and Migration of Hepatocellular Carcinoma Cells via Activation of ERK Signaling

**DOI:** 10.3390/ncrna7030052

**Published:** 2021-08-28

**Authors:** Qingchun Lu, Qian Guo, Mingyang Xin, Casey Lim, Ana M. Gamero, Glenn S. Gerhard, Ling Yang

**Affiliations:** Department of Medical Genetics and Molecular Biochemistry, Lewis Katz School of Medicine at Temple University, Philadelphia, PA 19140, USA; qingchun.lu@temple.edu (Q.L.); qian.guo0001@temple.edu (Q.G.); mia.xin@temple.edu (M.X.); casey.lim@temple.edu (C.L.); ana.gamero@temple.edu (A.M.G.); gsgerhard@temple.edu (G.S.G.)

**Keywords:** long non-coding RNA, TP53TG1, hepatocellular carcinoma, ERK pathway, oncogene

## Abstract

Long non-coding RNA (lncRNA) TP53 target 1 (TP53TG1) was discovered as a TP53 target gene. TP53TG1 has been reported as having dual roles by exerting tumor-suppressive and oncogenic activities that vary depending on the cancer type. Yet, the role of TP53TG1 in hepatocellular carcinoma (HCC) is not fully understood. In this study, we performed both gain- and loss-of-function studies to determine the biological role of TP53TG1 in HCC. We found that the knockdown of TP53 in HCC cells caused the upregulation of TP53TG1. Furthermore, we found that the knockdown of TP53TG1 not only suppressed HCC cell proliferation and migration, but also reduced intrinsic ERK signaling. In contrast, the overexpression of TP53TG1 increased ERK activation and enhanced HCC proliferation. In conclusion, our study reveals an oncogenic role of TP53TG1 in HCC, which provides a novel insight into the cell-type-specific function of TP53TG1 in HCC.

## 1. Introduction

Hepatocellular carcinoma (HCC) is a lethal malignancy that accounts for 75–80% of primary liver cancer cases [1]. It is also the fourth most common cause of cancer-related death worldwide [2]. HCC is frequently diagnosed late because it does not cause symptoms at the early stage. When diagnosed, patients have often progressed to an intermediate or advanced stage and are no longer eligible for locoregional treatment [3]. For these patients, systemic treatment is their only option that modestly increases the median overall survival by 3 to 6 months [4]. Consequently, there is an urgent need to gain further insight into the molecular mechanisms of HCC development and progression to identify potential therapeutic targets and prognostic markers.

In recent years, long non-coding RNAs (lncRNAs), a class of transcripts longer than 200 nucleotides without protein-coding potential, have been identified as important regulators in cancer via participating in a wide range of biological processes [5]. For instance, lncRNAs can regulate cancer cell proliferation, migration, and epithelial–mesenchymal transition (EMT) [6,7,8]. In addition, the aberrant expression of these cancer-related lncRNAs turns them into potential candidates for diagnostic and prognostic biomarkers in cancers [9]. Of note, several lncRNAs have been reported to be important regulators in HCC. As an example, the downregulation of lncRNA BCAR4 in the HCC cell line, HepG2, can suppress cell proliferation and invasion, as well as induce apoptosis [10]. Another lncRNA, HCP5, which is upregulated in HCC tissues and HCC cell lines, drives liver oncogenesis and expedites the progression of HCC by promoting HCC proliferation and metastasis [11]. LncRNA HAND2-AS1 also functions as an oncogene by promoting the self-renewal of liver cancer stem cells [12]. The multiple dimensions of lncRNA function suggest that lncRNAs are indispensable regulators in HCC development and progression.

The ERK signaling pathway regulates cancer by promoting cancer cell growth, survival, and motility [13]. In about 50% of human HCC tissues, increased levels of phosphorylated ERK (p-ERK, the activated form of ERK) were observed [14,15]. The activation of ERK signaling contributes to the development of HCC by activating a variety of transcription factors [13]. Inhibitors of the ERK pathway exert an antitumor effect by suppressing the proliferation and enhancing the apoptotic rate of HCC cells [13]. These studies underscore the important role of the ERK signaling pathway in HCC. Nonetheless, the molecular basis of the activation of the ERK signaling pathway in HCC remains elusive. Increasing evidence indicates that non-coding RNAs, including lncRNAs and microRNAs (miRNAs), play a critical role in ERK activation in cancer [16]. The identification of novel non-coding RNA regulators in the activation of the ERK signaling pathway in HCC may lead to the development of novel diagnostic and therapeutic strategies for HCC.

The lncRNA TP53TG1, first reported as a TP53-induced gene in human colon cancer [17], plays a highly cell- and tissue-specific role in cancer. For example, TP53TG1 has been found to serve as a tumor suppressor in human colorectal cancer, gastric cancer, and non-small cell lung cancer, whereas it functions as an oncogene in glioma and pancreatic ductal adenocarcinoma [18,19,20,21]. Because TP53 mutations are associated with HCC [22], and because of the important role of TP53TG1 in other cancers, we reasoned that lncRNA TP53TG1 may also be important in HCC. In this study, we found that TP53TG1 is negatively regulated by TP53 in HCC. TP53TG1 promoted the proliferation of HCC cells and their migration through the activation of ERK signaling. Our study has identified TP53TG1 as a novel key regulator of ERK signaling and an oncogene in HCC. 

## 2. Results

### 2.1. TP53TG1 Is Regulated by TP53 in HCC

**Figure 1:**
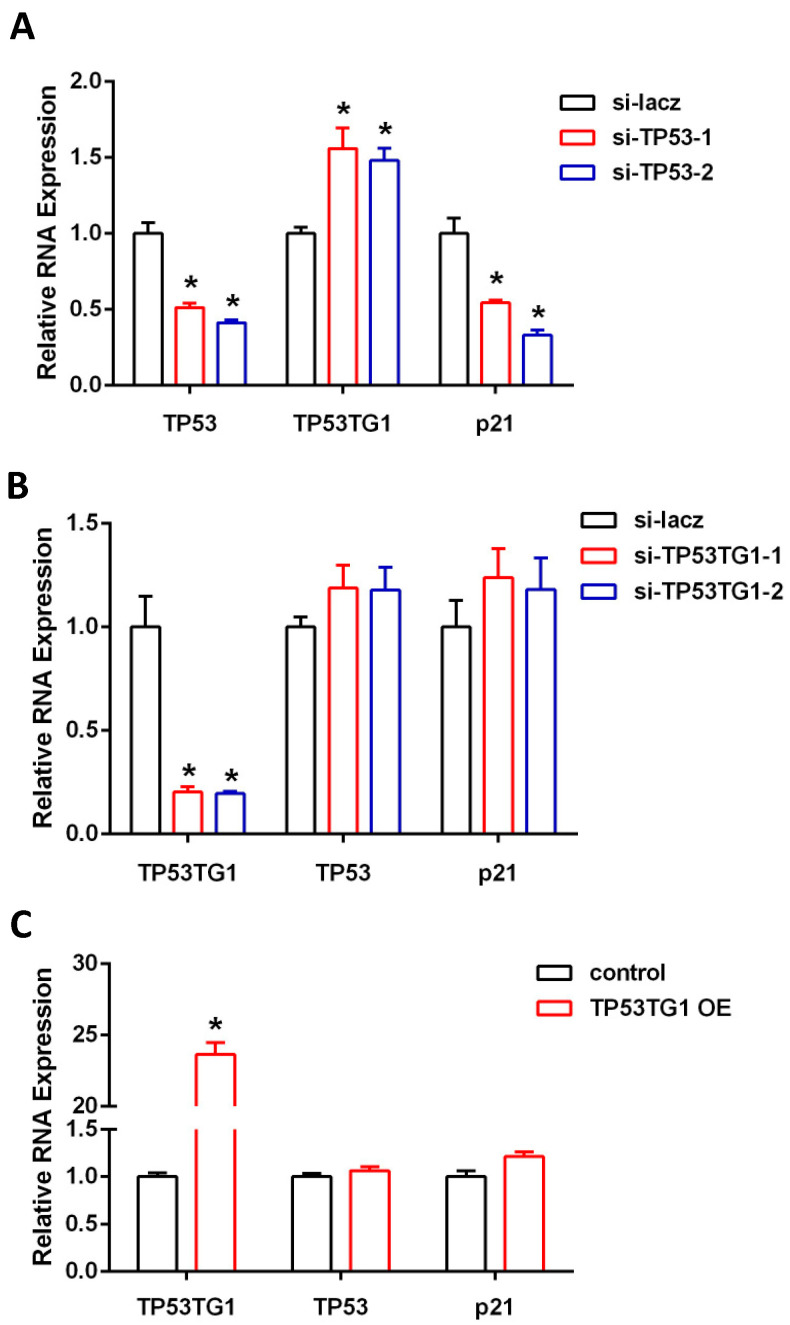
TP53TG1 is regulated by TP53 in HepG2. (**A**–**C**) qPCR analysis of TP53, TP53TG1, and p21 (normalized to 18S) in HepG2 cells transfected with lacz siRNA or TP53 siRNAs (**A**), HepG2 cells transfected with lacz siRNA or TP53TG1 siRNAs (**B**), and HepG2 cells transfected with control or TP53TG1 overexpression adenovirus (**C**). Error bars represent SEM, *n* = 3, * *p* < 0.05 (si-RNA vs. si-lacz).

TP53TG1 was reported to be regulated by TP53 in human colon cancer [17]. However, whether TP53TG1 is regulated by TP53 in HCC is not known. We first knocked down TP53 in HepG2, a TP53 wild-type HCC cell line, using two distinct siRNAs. As expected, TP53 knockdown (KD) downregulated P21 expression, a known downstream target of TP53 (Figure 1A). Interestingly, we found TP53TG1 to be upregulated in TP53 KD cells (Figure 1A). Next, we investigated whether TP53TG1 participated in a forward feedback loop in the TP53 pathway. We knocked down or overexpressed TP53TG1 in HepG2 cells and found that expression levels of TP53 and its downstream target P21 did not change (Figure 1B,C). These results indicate that TP53TG is negatively regulated by TP53 but TP53TG1 does not reciprocally affect the TP53 pathway in HCC.

### 2.2. Reducing TP53TG1 Decreases HCC Cell Proliferation and Migration 

**Figure 2:**
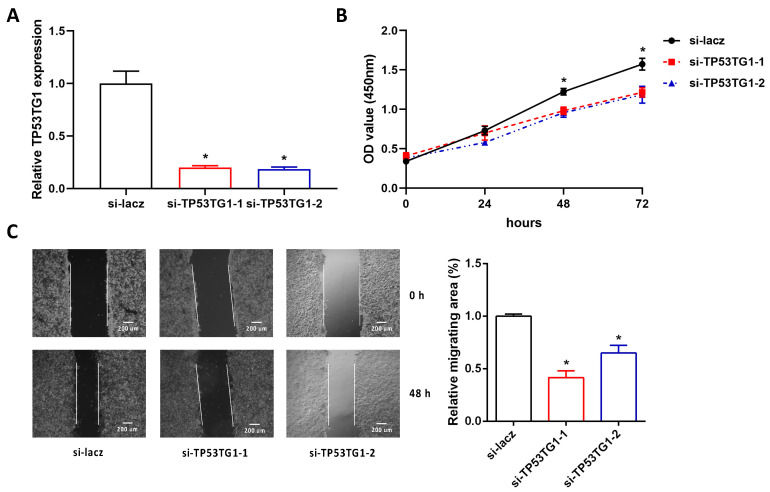
Knockdown of TP53TG1 suppresses cell proliferation and migration in HepG2. (**A**–**C**) The RNA expression level of TP53TG1 (normalized to 18S), *n* = 3 (**A**), proliferation rate, error bars represent SD, *n* = 6 (**B**) and migration rate, *n* = 3 (**C**) in HepG2 cells transfected with lacz siRNA or TP53TG1 siRNAs. * *p* < 0.05 (si-RNA vs. si-lacz).

**Figure 3:**
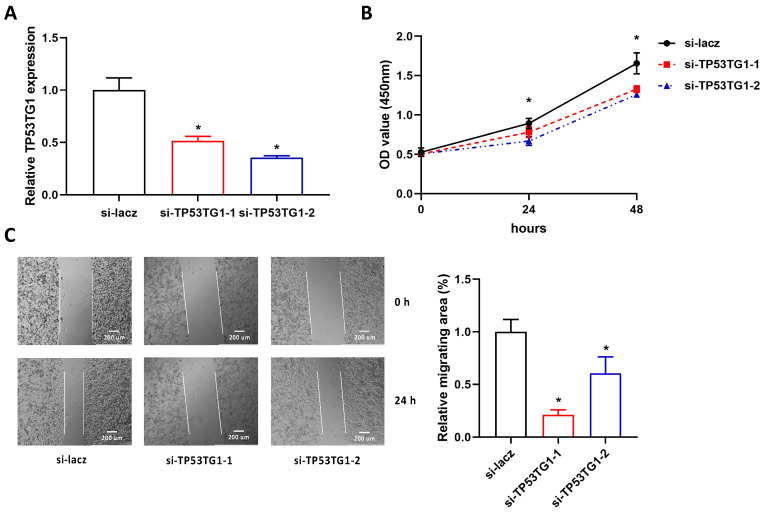
Knockdown of TP53TG1 suppresses cell proliferation and migration in PLC/PRF/5. (**A**–**C**) The RNA expression level of TP53TG1 (normalized to 18S), *n* = 3 (**A**), proliferation rate, error bars represent SD, *n* = 6 (**B**) and migration rate, *n* = 3 (**C**) in PLC/PRF/5 cells transfected with lacz siRNA or TP53TG1 siRNAs. * *p* < 0.05 (si-RNA vs. si-lacz).

To determine the functional role of TP53TG1 in HCC, we measured the cell proliferation and migration rate in TP53TG1 KD HepG2 cells. More than 70% of TP53TG1 expression was reduced by TP53TG1 siRNAs compared to the si-lacz control groups (Figure 2A). Next, the proliferation and migration rates of HepG2 cells were determined by Cell Counting Kit-8 (CCK-8) assay and wound-healing assay, respectively. Our results showed that KD of TP53TG1 suppressed HepG2 cell proliferation and migration (Figure 2B,C). In addition, we performed the same experiments in another HCC cell line PLC/PRF/5, a TP53 mutant cell line. Consistently, KD of TP53TG1 suppressed PLC/PRF/5 cell proliferation and migration (Figure 3A–C). These results suggest that TP53TG1 works as an oncogene in HCC. 

### 2.3. TP53TG1 Promotes EKR Signaling Activation in HCC

**Figure 4:**
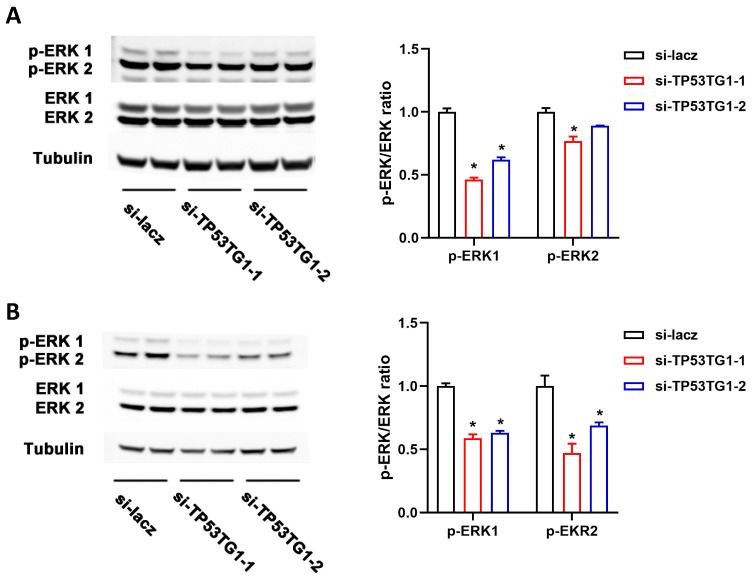
Knockdown of TP53TG1 inhibits ERK signaling. (**A**,**B**) Protein expression level (left) and quantification (right) of p-ERK1/p-ERK2 and ERK1/ERK2 in HepG2 cells (**A**) or PLC/PRF/5 cells (**B**) transfected with lacz siRNA or TP53TG1 siRNAs. The protein expression levels of p-ERK1 and p-ERK2 were normalized to ERK1 and EKR2, respectively. Error bars represent SEM, * *p* < 0.05 (si-RNA vs. si-lacz).

**Figure 5:**
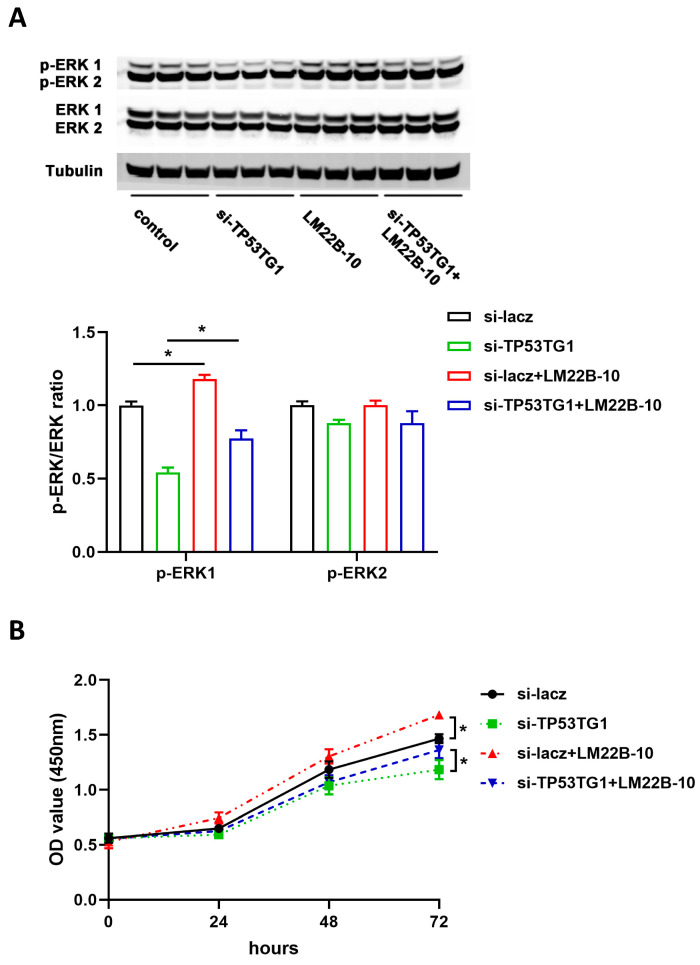
TP53TG1 works through the ERK pathway. (**A**,**B**) Protein expression level (top) and quantification (bottom) of p-ERK1/p-ERK2 and ERK1/ERK2 (**A**), and cell proliferation rate, error bars represent SD, *n* = 6 (**B**) in HepG2 cells treated with si-lacz, si-TP53TG1, si-lacz + LM22B-10, or si-TP53TG1 + LM22B-10. The protein expression levels of p-ERK1 and p-ERK2 were normalized to ERK1 and EKR2, respectively. * *p* < 0.05.

**Figure 6:**
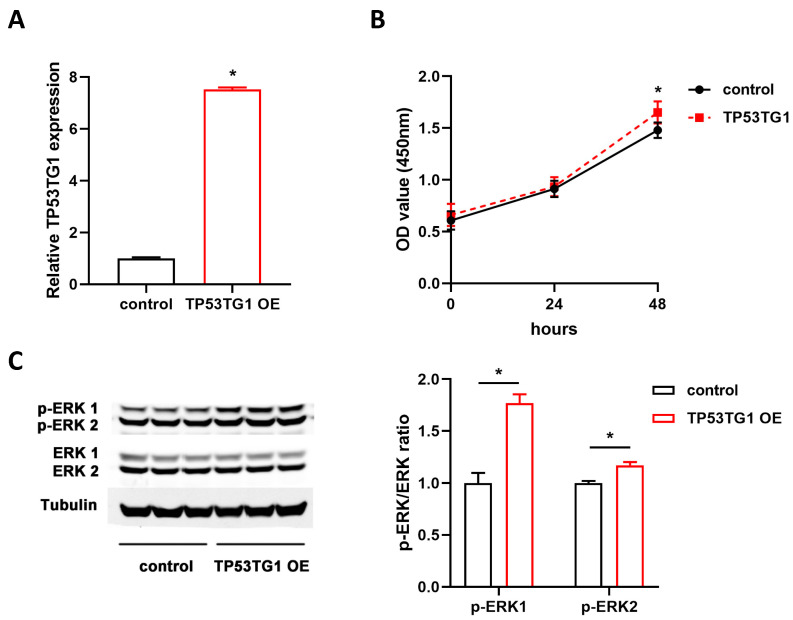
Overexpression of TP53TG1 increases cell proliferation and ERK activation in HepG2. (**A**–**C**) The RNA expression level of TP53TG1 (normalized to 18S), *n* = 3 (**A**), proliferation rate, error bars represent SD, *n* = 6 (**B**), and protein expression level of p-ERK1/p-ERK2 and ERK1/ERK2 (left) as well as qualification (right) (**C**) in HepG2 cells transfected with control or TP53TG1 overexpression adenovirus. The protein expression levels of p-ERK1 and p-ERK2 were normalized to ERK1 and EKR2, respectively. * *p* < 0.05.

ERK signaling activation plays a critical role in the pathogenesis of HCC. To determine whether TP53TG1 altered ERK signaling, we examined the level of activated ERK (p-ERK, phosphorylated ERK) and total ERK in HepG2 and PLC/PRF/5 cells transfected with either si-lacz or si-TP53TG1 (a pool of two siRNAs, si-TP53TG1-1 and si-TP53TG1-2). The results showed that TP53TG1 KD dramatically inhibited ERK1/ERK2 activation without affecting total ERK1/ERK2 levels (Figure 4A,B). To further determine whether TP53TG1 regulated HCC proliferation through ERK, we performed a rescue experiment by which cells were exposed to LM22B-10, an ERK activator. As shown in Figure 5A, TP53TG1 KD reduced p-ERK1/p-ERK2 levels. The addition of LM22B-10 was able to restore p-ERK1 levels in the TP53TG1 KD cells. Consistent with this observation, the treatment of TP53TG1 KD cells with LM22B-10 rescued cell proliferation (Figure 5B). Reciprocally, TP53TG1 overexpression increased cell proliferation and ERK1/ERK2 activation (Figure 6A,B). Collectively, these results denote that TP53TG1 plays an oncogenic role and functions through the activation of ERK in HCC.

## 3. Discussion

LncRNAs have emerged as critical regulators in a variety of cancers. Recently, much attention has been directed at exploring their roles and underlying molecular mechanisms in the pathogenesis of HCC. In the present study, we found that the expression level of lncRNA TP53TG1 was negatively regulated by TP53 and that KD of TP53TG1 reduced HCC cells’ proliferation and migration significantly. Moreover, our results revealed that TP53TG1 exerted its function through the ERK pathway. These findings suggest that lncRNA TP53TG1 has tumorigenic activity in HCC.

TP53TG1 was first identified as a downstream target of TP53 in colon cancer [17]. In the present study, we found that the knockdown of TP53 led to the upregulation of TP53TG1 in HCC. This result is consistent with our findings that TP53TG1 promotes HCC cell proliferation and migration. A previous study suggested that TP53TG1 may play an important role in the TP53 signaling pathway [18]. We found that neither TP53TG1 downregulation nor overexpression affected the expression level of TP53 and its downstream target p21. Our results indicate that TP53TG1 is regulated by TP53 but may not have a feedback role in the TP53 signaling pathway in HCC.

It is unclear how TP53TG1 regulates ERK signaling in HCC. KRAS is a member of the RAS family and is a known upstream regulator of ERK1/2 [23]. Upon activation, KRAS interacts with RAF kinase and promotes its phosphorylation, which in turn activates MEK kinases and sequentially phosphorylates and activates ERK1/2 [24,25]. In pancreatic ductal adenocarcinoma (PDAC), TP53TG1 works via the miR-96/KRAS axis through directly binding to tumor suppressor miR-96 and preventing miR-96 from binding to its target oncogene KRAS, thus elevating KRAS expression [20]. However, unlike the tumor suppressive role of miR-96 in PDAC, miR-96 functions as an oncogenic miRNA that inhibits apoptosis and promotes the proliferation, migration, and invasion of HCC [26,27]. Therefore, miR-96 is unlikely to target KRAS in HCC, since miRNAs negatively regulate target genes while both KRAS and miR-96 work as oncogenes in HCC. Future studies should seek to elucidate the mechanism by which TP53TG1 activates ERK signaling. 

Like many other lncRNAs, the roles of TP53TG1 are highly cell- and tissue-specific [18,19,20,21]. In gastrointestinal cancer cells, TP53TG1 plays a tumor suppressive role through the sequestration of the RNA binding protein YBX1, thus blocking its oncogenic activity [18]. TP53TG1 also improves the sensitivity of the anti-tumor drug cisplatin in non-small cell lung cancer by interacting with the miR-18a/PTEN axis [19]. On the contrary, TP53TG1 promotes tumor growth by binding to miR-96 to enhance KRAS activation in PDAC [20]. TP53TG1 can also function as a sponge of miR-524 and exert an oncogenic role in glioma [21]. Furthermore, TP53TG1 may promote glioma cell proliferation and migration through affecting glucose metabolism [28]. Here, we found that TP53TG1 is involved in the proliferation and migration of HCC cells through the activation of ERK signaling. However, our observations differ from a recent study showing that TP53TG1 works as a tumor suppressor gene in HCC through the inactivation of WNT/β-catenin signaling [29]. This discrepancy may be attributable to the highly cell-type-specific roles of TP53TG1: we used HepG2 (HCC cell line) and PLC/PRF/5 (HCC cell line), while they used Huh7 (HCC cell line), SK-Hep1 (liver sinusoidal endothelial cell line), and HCCLM3 (cell line from lung metastatic lesions of HCC). In addition, the underlying cause of HCC may be important. The P53 pathway was found to be upregulated in HBV-associated HCC [30], while protein levels were decreased in NASH-related HCC but not HCV-related [31]. Future studies into how TP53TG1 functions in a cell-type-specific manner will be critical to determine the contribution of TP53TG1 to the pathogenesis of HCC and other types of cancer. 

In summary, our data show for the first time that lncRNA TP53TG1 is regulated by TP53 and can promote cell proliferation and migration by activating ERK signaling in HCC. This study provides a novel insight into the role and mechanism of TP53TG1 in HCC.

## 4. Materials and Methods

### 4.1. Cell Culture 

Authenticated and mycoplasma-free human HCC cell lines HepG2 and PLC/PRF/5 were obtained from the Cell Culture Facility at Fox Chase Cancer Center. HepG2 cells were cultured in DMEM medium (Thermo Fisher Scientific, USA) containing 10% cosmic calf serum (Hyclone Laboratories Inc., USA) and 1% penicillin–streptomycin (Thermo Fisher) in an incubator with 5% CO_2_. PLC/PFR/5 cells were cultured in RPMI-1640 medium (Thermo Fisher) with 10% cosmic calf serum and 1% penicillin–streptomycin in an incubator with 5% CO_2_. 

### 4.2. Cell Transfection

Small interfering RNAs (siRNAs) complementary to the TP53TG1, TP53, and lacz sequences were synthesized by Sigma. The siRNA sequences were as follows: si-TP53TG1-1-F, 5′-CAAACUGUUUGGAAAGCUA-3′; si-TP53TG1-1-R, 5′-UAGCUUUCCAAACAGUUUG-3′; si-TP53TG1-2-F, 5′-CUUCCCUCUUAAUGAAUAA-3′; si-TP53TG1-2-R, 5′-UUAUUCAUUAAGAGGGAAG-3′; si-TP53-1-F, 5′-GAGGAUUUCAUCUCUUGUA-3′; si-TP53-1-R, 5′-UACAAGAGAUGAAAUCCUC-3′; si-TP53-2-F, 5′-CGGCGCACAGAGGAAGAGA-3′; si-TP53-2-R, 5′-UCUCUUCCUCUGUGCGCCG-3′; si-lacz-F, 5′-CUACACAAAUCAGCGAUUU-3′; si-lacz-R, 5′-AAAUCGCUGAUUUGUGUAG-3′. siRNAs were transfected into cells using Lipofectamine RNAiMAX (Thermo Fisher) according to the manufacturer’s instructions. For TP53TG1 overexpression experiments, empty adenovirus and adenovirus-carrying TP53TG1 were generated. Cells were infected with adenovirus at a multiplicity of infection (MOI) of 50 for 24 h.

### 4.3. Cell Proliferation Assay

The Cell Counting Kit-8 (CCK-8; Dojindo, JPN) reagent was used to measure cell proliferation. Twenty-four hours after transfection, 1.5 × 10^4^ of HepG2 cells or 5 × 10^3^ PLC/PRF/5 cells were harvested and seeded into 96-well plates. After another 0, 24, 48, or 72 h, 10 μL of CCK-8 reagent was added to each well and incubated for 2 h. Absorbance of the cells was measured using SpectraMax i3 at 450 nm.

### 4.4. Cell Migration Assay

Cell migration was assessed by a wound-healing assay [32]. Cells were seeded into twelve-well plates and transfected for 48 h. When cells reached 100% confluence, a p200 pipet tip was used to make a scratch. Then, cells were washed with PBS and cultured in a serum-free medium. Cells were imaged by a light microscope 24 h or 48 h later after scratching.

### 4.5. Western Blot Assay

Cells were lysed in RIPA buffer (Cell Signaling Technology) containing protease and phosphatase inhibitors (Thermos Scientific). A total of 40 μg of protein was separated on a gradient gel and transferred to a polyvinylidene difluoride (PVDF) membrane. Next, PVDF membranes were incubated with primary antibodies against ERK (Cell Signaling Technology, 1:1000), p-ERK (CST, 1:1000), and tubulin (CST, 1:1000) overnight. After several washes with phosphate-buffered saline with Tween 20 (PBST), membranes were incubated with secondary antibody (LI-COR Biosciences, 1:5000) and visualized by a quantitative fluorescence imaging system (LI-COR).

### 4.6. RNA Extraction, Reverse Transcription, and qRT-PCR

Total RNA from cells was extracted using Trizol Reagent (Thermo Fisher) and reverse transcribed to cDNA using a RevertAid RT Reverse Transcription kit (Thermo). qRT-PCR was performed using SYBR Green (Thermo). The following primer sequences were used: TP53TG1-F, 5′-ACGAAGGTACCCAACCCTCT-3′; TP53TG1-R, 5′-TGTTCTTTTGCCAAGACACG-3′; TP53-F, 5′-GTGACACGCTTCCCTGGATT-3′; TP53-R, 5′-TGTTTCCTGACTCAGAGGGG-3′; p21-F, 5′-TTAGCAGCGGAACAAGGAGT-3′; p21-R, 5′-GCCGAGAGAAAACAGTCCAG-3′; 18S-F, 5′-AGTCCCTGCCCTTTGTACACA-3′; 18S-R, 5′-CGATCCGAGGGCCTCACTA-3′. 18S was used as an internal control. Change in gene expression level was determined using the 2^−ΔΔCt^ method.

### 4.7. Statistical Analysis

A two-tailed unpaired Student’s t-test was used for comparison between groups. A one-way analysis of variance (ANOVA) followed by Bonferroni or Dunnett post hoc test was performed for multiple comparisons. All data were presented as mean ± SEM unless specified otherwise. *p* < 0.05 was considered to be statistically significant. 

## Data Availability

Not applicable.
